# Delayed Epistaxis which Was Developed after Orthognathic Surgery with Le Fort I Osteotomy and Managed by Endoscopic Cauterization

**DOI:** 10.1155/2022/3057472

**Published:** 2022-02-22

**Authors:** Yuya Kurasawa, Hitoshi Sato, Ryogo Katada, Takanobu Inada, Tatsuo Shirota, Toshikazu Shimane

**Affiliations:** ^1^Department of Oral and Maxillofacial Surgery, Division of Oral Oncology, Showa University, School of Dentistry, Tokyo, Japan; ^2^Department of Oral and Maxillofacial Surgery, Showa University, School of Dentistry, Tokyo, Japan

## Abstract

A case of delayed epistaxis from the mucosa behind the right side of the inferior nasal mucosa 11 days after orthognathic surgery by Le Fort I osteotomy is presented. The patient was a 31-year-old man who underwent orthognathic surgery under general anesthesia. No abnormal findings were found during or after the operation. The patient was discharged from the hospital 10 days postoperatively. However, bleeding from the right nasal cavity occurred suddenly on the night after discharge, and he presented to our hospital again. The epistaxis was stopped once by nasal packing containing 0.001% epinephrine and systemic infusion of carbazochrome sulfonic acid and tranexamic acid. However, when the nasal packing was removed the next day, right nasal epistaxis was observed again. Curvature of the nasal septum and thickening of the inferior turbinate mucosa were seen on inspection; although, no active bleeding point was identified. Decreased nasal mucosa thickening and bleeding were observed after nasal packing containing 0.02% epinephrine. When the inside of the nasal cavity was observed endoscopically, an approximately 2 mm laceration was found in the mucosa behind the side wall of the right inferior nasal mucosa, and bleeding from the same part was confirmed. After endoscopic cauterization for hemostasis of the nasal mucosa, no rebleeding was observed. Although delayed epistaxis after Le Fort I osteotomy are often performed CT angiography to confirm the bleeding site, endoscopic cauterization would be primarily useful because of less invasiveness.

## 1. Introduction

The Le Fort I osteotomy to correct maxillary deformities is a versatile technique that avoids complications. The osteotomy design has undergone modification to enhance the ability of the surgeon to accurately reposition the maxilla and to improve bony contact and thus the initial stability of the mobilized jaw [1, 2]. Even though it is a simple and reliable procedure after years of clinical use, it is not completely free of complications [3, 4]. Although most complications are fortunately nonfatal, major intraoperative and postoperative bleeding that is potentially life-threatening may occur. The authors present a case of delayed, massive epistaxis caused by a ruptured nasal mucosa as a postoperative complication of Le Fort I osteotomy.

## 2. Case Presentation

**Figure 1:**
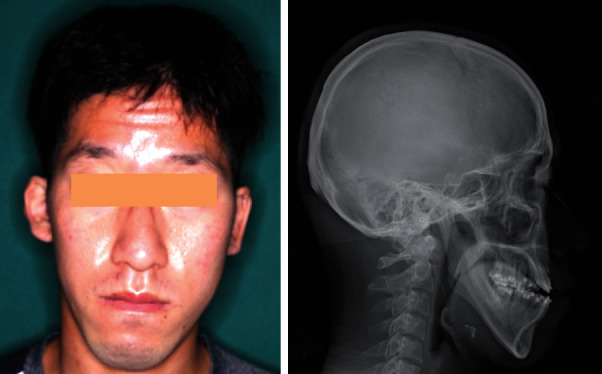
Preoperative facial photograph and lateral cephalometric radiograph. The mandible is deviated to the right, with anterior crossbite.

**Figure 2:**
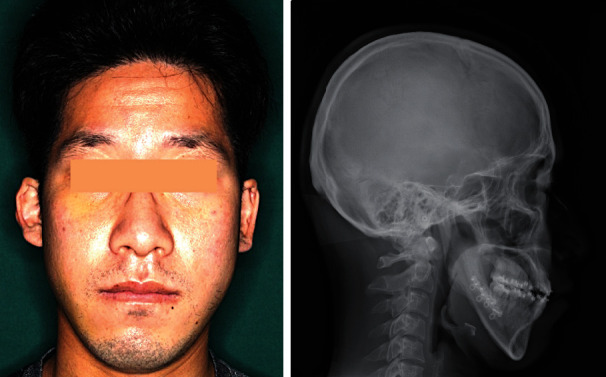
Postoperative facial photograph and lateral cephalometric radiograph. Mandibular deviation and occlusal relationships are improved.

A 31-year-old Japanese man (height, 164.0 cm; weight, 63.5 kg) with a chief complaint of an occlusal abnormality began treatment in the orthodontic department of our institution in November 2017. His personal and family medical histories were unremarkable. The diagnosis was facial asymmetry with class III malocclusion accompanied by skeletal mandibular prognathism (Figure 1). The frontal cephalometric X-ray showed the left upper and lower first molars positioned about 2 mm lower than those on the right, and the occlusal plane inclined upwards, toward the right. Three-dimensional skeletal analysis showed the left upper first molar positioned about 1 mm more forward than the right upper first molar. Thus, orthognathic surgery, including Le Fort I osteotomy and bilateral sagittal split ramus osteotomy, was scheduled for August 2020 after the patient completed presurgical orthodontic treatment. His nutritional status was good, and laboratory findings three weeks before surgery showed no abnormalities. Le Fort I osteotomy and bilateral sagittal split ramus osteotomy proceeded under general anesthesia in August 2020. The left first molar region was elevated 2.5 mm, and the maxilla was rotated with the right first molar as the center. The mandible was repositioned posteriorly (right and left, 5.0 and 2.0 mm, respectively). Bilateral descending palatine arteries were exposed by removing interfering bone around them. The maxillary tuberosity was also carefully removed with an ultrasonic vibration cutting device. The volume of intraoperative blood loss was 155 mL, and surgical duration was 4 h and 54 min. Osteosynthesis was performed with bioabsorbable plates for the maxilla (Super Fixsorb, Teijin, Tokyo, Japan) and titanium plates for the mandible (MOJ system, Johnson & Johnson, Tokyo, Japan). The patient was free of adverse events and abnormalities and was discharged 10 days after surgery (Figure 2).

The night after discharge, he presented to our hospital again with epistaxis from the right side. The patient was hemodynamically stable. Laboratory investigations were normal, with a hemoglobin of 12 g/dl. After readmission to our hospital, bilateral anterior nasal packing containing 0.001% epinephrine was performed. In addition, systemic infusion of carbazochrome sulfonic acid and tranexamic acid was performed. The bleeding was controlled.

**Figure 3:**
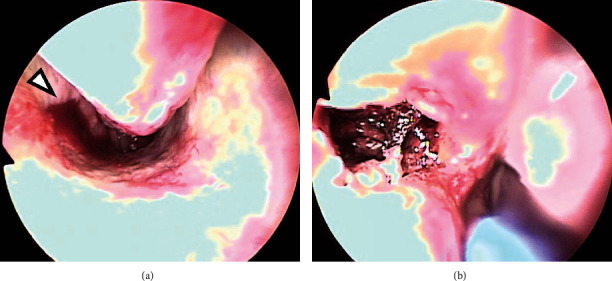
Endoscopic image ((a) at the time of bleeding, (b) at the time of hemostasis). (a) A laceration is found on the side wall of the right inferior nasal passage, and arterial bleeding is observed (arrowhead). (a) Hemostasis is achieved by cauterization of the nasal mucosa.

**Figure 4:**
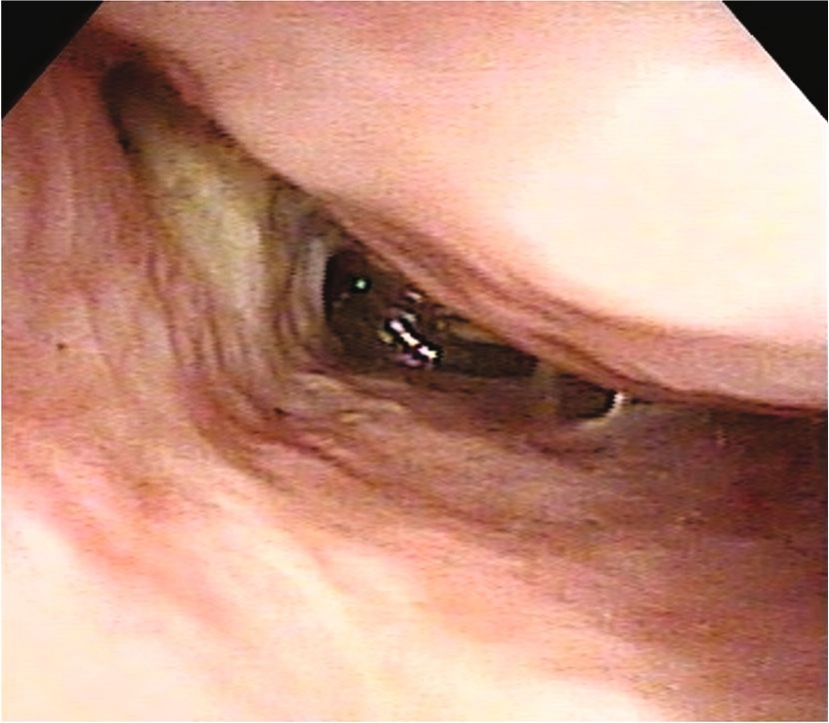
Endoscopic image (7 days after endoscopic cauterization hemostasis). Scarring of the nasal mucosa at the bleeding point is observed.

On the second day of readmission, continuous posterior nasal bleeding was noted when the nasal packing was removed. The complete blood count showed that the hemoglobin had decreased to 10.6 g/dl. Inspection of the right nasal cavity showed protrusion of the nasal septal cartilage and thickening of the inferior turbinate mucosa. However, the bleeding site could not be confirmed. Bleeding became weaker with insertion of nasal packing containing 0.02% for about 15 min, and a rigid endoscope (Olympus, Tokyo, Japan) could be inserted. On endoscopy, a laceration of about 2 mm was found in the mucosa behind the side wall of the right inferior nasal passage, and arterial bleeding from the same part was confirmed (Figure 3(a)). Therefore, cauterization was performed with a suction coagulator for hemostasis while visually observing the bleeding site under an endoscope, and an alginate wound dressing (Sorbsan®, ALCARE Co., Ltd., Tokyo, Japan) was inserted into the cauterization site to complete the hemostasis treatment (Figure 3(b)). Subsequently, there was no further epistaxis, and the patient was discharged on the day after the cauterization procedure for hemostasis. Seven days after the procedure, the scarred tissue on the bleeding site was seen endoscopically (Figure 4). Since then, regular follow-up has continued, with no remarkable adverse events, including no unstoppable epistaxis.

## 3. Discussion

It has been reported that complications following Le Fort osteotomy are relatively rare compared to the number of procedures performed [5]. Fortunately, most of the complications are not severe and/or fatal. Politis noted that only two cases of life-threatening bleeding were reported following 750 Le Fort I osteotomies [6]. On the other hand, the article mentioned that such complications could be underestimated, since patients experiencing massive nasal bleeding are not necessarily treated in the same hospital or by the same surgeon.

During Le Fort I osteotomies, pterygomaxillary separation leads to damage to the descending palatine artery and pterygoid venous plexus. A tomodensitometric study to measure the position of the maxillary artery in relation to pterygomaxillary junction shows that there is a risk of injuring the artery around 20 mm above the inferior extremity of the pterygomaxillary junction [7]. Thus, to avoid bleeding complications during or after surgery, care is needed when performing pterygomaxillary separation. An ultrasonic bone curette is useful for a safe procedure when performing pterygomaxillary separation without damaging the surrounding tissues [8]. In the present case, an ultrasonic device was also used to perform procedures in the pterygomaxillary area to safely mobilize the pterygoid process, and the postoperative bleeding was not from this area. Recently, a large clinical trial for down fracture in Le Fort I osteotomy by digital pressure alone showed no severe complications [9]. Moreover, there are no strong evidences of the usefulness of osteotomes in pterygomaxillary disjunction or disjunction via the maxillary tuberosity [9]. Thus, pterygomaxillary separation without an osteotome such as a bone chisel should be considered to avoid bleeding complications.

In general, vessel injury causes acute bleeding that should be controllable intraoperatively. However, in a case with partial vessel injury, vulnerability of the vessel wall can lead to the formation of a pseudoaneurysm [10]. It has been reported that the cause of delayed epistaxis in Le Fort I osteotomy may be rupture of a pseudoaneurysm [10–14]. The epistaxis in the present case was managed by endoscopic cauterization, achieving hemostasis for a laceration of the nasal mucosa. Thus, the cause of the epistaxis might have been a pseudoaneurysm and/or postoperative traumatic injury to a small artery on the nasal mucosa. Although most patients with a pseudoaneurysm develop postoperative bleeding within 14 days postoperatively, longer delays of up to 3 months postoperatively have been reported [14]. The incidence of this complication is rare, and more cases need to be studied to determine the best approach for diagnosis and treatment. Angiography confirms the diagnosis, precisely locating the site of bleeding, and treatment can be provided immediately if necessary. Thus, it was used in many previous reports with delayed epistaxis after the Le Fort I osteotomy were used it. However, CT angiography would take time and have the risk of unnecessary radiation exposure. Endoscopically confirmation of the bleeding site should be primarily performed the delayed epistaxis after the orthognathic surgery, because of the convenience and noninvasive procedure of it. In the management of the present case, it was elected not to perform embolization because hemostasis was achieved with endoscopic cauterization.

## 4. Conclusion

We described a case with delayed epistaxis after Le Fort I osteotomy which was managed by endoscopic cauterization. Although CT angiography might be useful to confirm the bleeding site, careful endoscopic examination should be performed prior to that in order to avoid the unnecessary radiation exposure.

## Data Availability

The data used to support the findings of this study are available from the corresponding author upon request.
